# Otoneurologic Evaluation in children with school difficulties: vestibular function investigation

**DOI:** 10.1016/S1808-8694(15)31177-0

**Published:** 2015-10-19

**Authors:** Eloisa Sartori Franco, Ivone Panhoca

**Affiliations:** 1 Master's degree in speech therapy, PUC - SP. Teacher in the Speech Therapy course, Piracicaba Methodist University - UNIMEP; Supervisor of audiology trainees - UNIMEP; 2 Doctoral thesis in sciences, Language Studies Institute - UNICAMP. Teacher in the Speech Therapy School - PUC, Campinas. Official external advisor in the “Child and Adolescent Health” program, Pediatrics Department, FCM/UNICAMP

**Keywords:** school difficulties, labyrinth diseases, vertigo

## Abstract

According to the literature, child vestibular dysfunctions can considerably affect their ability of communication and school performance.

**Aim:**

to study the vestibular function in children with school difficulties and associated symptoms. **Case study**: Clinical study with transversal cohort. **Method**: Research subjects were 50 school children between 7 and 12 years old, from a public school of Piracicaba city, during the years 2004 and 2005. The procedure was based on: anamnesis; otorrinolaryngologic evaluation; audiologic evaluation and vestibular evaluation.

**Results:**

All children assessed, 62.0% did not have school difficulties and 38.0% had school difficulties. Dizziness was the most common general complaint (36.0%). Migraine was the most common symptom regarding the school environment (50.0%). We found a high rate of normal vestibular condition (74.2%) in children without school difficulties and low normality rate in those with school difficulties (31.6%). All found vestibular alterations, both unilateral and bilateral, had been of peripheral irritative origin, accounting for 68.4% of cases for children with school difficulties and 25.8% for children without school difficulties.

**Conclusion:**

Dizziness, nausea, reading and copying difficulties presented a statistically significant relationship between the studied variables. All found vestibular alterations had been of peripheral irritative origin. Data showed a statistically significant relationship among variables.

## INTRODUCTION

The etiology of learning disabilities is varied and may involve organic, intellectual/cognitive and emotional factors or insufficient/inappropriate instruction; usually these factors interact.^1^, ^2^, ^3^ Increased knowledge about the neurobiology of language development and learning processes will certainly add to knowledge about the etiology. Systematic and accurate investigation of the diagnosis may direct health professionals about the best treatment for each case.^4^

Farias et al.^5^ assessed 103 children with and with no learning disabilities, of which 60 children (58.2%) reported no difficulties at school and 43 children (41.7%) presented learning disabilities.

Posture, balance and motor coordination have been recognized as important foundations for learning in general, including spoken and written language. The function of the vestibular system (with the central nervous system) is to control body position, eye movement and spatial perception; this system, therefore, is believed to have a significant influence on child development.^6^,^7^

Vertigo and dizziness, nausea, vomiting, falls, unbalanced or non-linear gait, headaches or ill-defined malaise, associated or not with visual distortion, excessive weariness, sudden behavioral changes, agitation, sleep disorders and fear of heights, may suggest vestibular system involvement.

Vestibular dysfunction in children may considerably affect communication abilities, the psychological state and school performance; thus, poor school functioning may be a valuable indicator of labyrinth disease.^6^

Early recognition of child vestibular diseases and etiological treatment are essential for preventing impaired motor development and poor language acquisition. Vestibular testing should be done in every child in which vestibular dysfunction is suspected, although it may not be easy to obtain a clear description of symptoms from the child or parents.^8^

Lavinsky et al.^9^ defined the criteria for identifying labyrinth diseases in children, as follows: the child does not like to be moved; poor school performance; frequent falls; unexplained nausea and vomiting; oral and written language acquisition disabilities; a history of migraine; use of ototoxic agents; and recurring acute otitis media.

Impaired coordination of movement and imprecise spatial perception of oneself and surrounding objects interfere with learning in children that have labyrinth disease. Inaptitude for certain physical movements, use of abnormal head positions while writing, distorted perception of size, weight, bodily structure, the size of distant objects and their spatial relation, alter the contact that children make with their environment, which negatively affects their physical and psychic development.^8^

Dizziness is frequent, although it is usually well tolerated, so that the complaint only arises in direct questioning. These children tend to be unquiet due to constantly seeking comfortable and safe positions; this leads to concentration difficulties and dispersion. They may not like playing or riding bicycles (or having the lateral support wheels removed), balancing on top of walls or curbs, skipping or using playground toys.^10^

Vestibular changes in dyslexic children led authors Frank and Levinson^11^ to raise the hypothesis that vestibular dysfunction and spontaneous nystagmus could interfere with sequential ocular fixation, which is needed for reading.

Quirós^12^ found that vestibular system changes in children could lead to speech disability and delayed motor development; this author underlined the need for careful assessments of school children, followed by an intervention program, if necessary.

Horak et al.^13^ studied vestibular function in children with and with no reading and writing disabilities and found altered oculo-vestibular reflexes in 20% of the children that had learning disabilities.

According to Hoyt,^14^ reading requires that saccadic movements and fixation periods alternate. Saccades run along 8 to 10 words, alternating with ocular fixation periods, and ending with a long saccade in order to start another line.

According to Caovilla et al.,^8^ benign paroxysmal vertigo is one of the most frequent childhood labyrinth diseases; it is characterized by bouts of dizziness or altered balance that are commonly associated with language disabilities, psychological behavioral alterations and poor school performance.

Ganança^15^ investigated 64 children with language disabilities and no complaints of dizziness, and found a high incidence of peripheral topodiagnostic vestibular alterations.

Oculomotor integrity and intact vestibular interconnections are needed to follow the teacher in the classroom within the child's visual field, to make copies, to transcribe lessons written on the blackboard, to read books, to write and to concentrate.

Non-specific complaints such as sudden behavioral changes, agitation, sleep disorders, headaches, fear of heights, fear of darkness, falls, nausea and vomiting should be valued (together with classic labyrinthic symptoms such as rotating and non-rotating dizziness, dysacusis, tinnitus, auricular fullness, low tolerance for loud sounds, etc.). These signs and symptoms may be the evidence of problems that will result in poor school performance and impaired development in general. These findings are due to vestibular system disorders and their relation to the central nervous system, vision, proprioception, as well as various other organs and systems frequently at a distance from the labyrinth.

This study aimed to investigate vestibular function in school age children that presented learning disabilities, by recording their vestibular complaints. It was expected that by better understanding such vestibular disorders and their causes, further studies could be done to promote early intervention programs.

## MATERIAL AND METHOD

### Type of study

The Research Ethics Committee approved this experimental study (protocol number 423/2003), which was undertaken in the Teaching Clinic of a university in the state of Sao Paulo.

### Case selection

Subjects were children aged between 7 and 12 years that attended public schools in the city of Piracicaba; they were invited to the Teaching Clinic between 2004 and 2005.

We assessed separately two groups of children; one of them had no learning disabilities and the other complained of difficulties at school. These groups were formed based on clinical histories provided by the children and their parents and/or caretakers.

For this paper 50 children and their parents were invited to the Teaching School to learn about and to participate in the study.

A meeting at a set date, time and place was held to distribute a letter of information and a free informed consent form to each participant. Consultations were scheduled following parent/caretaker approval. All of the subjects signed the free informed consent form for the investigation and for publishing the data, according to the regulation number 196/96.

• inclusion criteria - group with complaints of difficulties at school: children that reported learning difficulties; pure tone thresholds between 500Hz to 8000Hz below or equal to 15 dBNA;^16^,^17^ type A tympanometry, and contralateral and ipsilateral acoustic reflexes present bilaterally at 500Hz to 4000Hz.^18^
• inclusion criteria - group with no learning disabilities: children with no complaints suggesting learning disabilities; pure tone thresholds between 500Hz to 8000Hz below or equal to 15 dBNA;^16^,^17^ type A tympanometry and contralateral and ipsilateral acoustic reflexes present bilaterally at 500Hz to 4000Hz.^18^
• exclusion criteria - both groups: children aged below 7 years and over 12 years; children that reported auditory and visual symptoms or alterations that might interfere with the results of this investigation.

### Procedures

 

### Clinical history:

The clinical history was taken from all children and their parents; information included vestibular complaints, particularly vertigo, and associated complaints such as other auditory findings, neurovegetative symptoms and neurological cases where involvement of the posterior fossa was suspected.

### Otorhinolaryngological exam:

An ENT specialist volunteered to carry out otorhinolaryngological examinations of both groups; the aim was to exclude ear, nose and throat disorders that might affect the auditory and vestibular systems.

### Audiological testing:

Audiological testing was composed of pure tone audiometry (air and bone conduction, if needed), investigation of the speech recognition percentage index, of the speech recognition threshold (SRT) and acoustic immitance testing, according to Mangabeira Albernaz et al.'s^18^ criteria. Pure tone and voice audiometry were carried out in an acoustic booth, using a MADSEN MIDIMATE 622 audiometer and a MADSEN ZO-72 middle ear analyzer.

Glorig and Davis's^16^ and Mangabeira Albernaz et al.'s^17^ criteria were adopted; these take into account normal hearing standards at various ages to characterize normal hearing limits.

These results were used only as inclusion criteria.

### Vestibular examination:

Children were instructed to abstain from coffee, tea, chocolate or any labyrinth stimulant medication during 72 hours prior to the examination.

The vestibular examination was done according to the orientation given by Caovilla et al.^19^ for the sequence of testing and the vestibular exam interpretation parameters. Interpretation of results was done according to Ganança et al.'s^20^ parameters.

Vectonystagmography was done using a three-channel digital computerized vectonystagmograph (VECWIN system), a visual stimulator (model - EVR 03) and an air otocalorimeter (model - NGR 05, NEUROGRAFF ELETRO-MEDICINA LTDA).

**The following tests were done**:

**Investigation of nystagmus or positional vertigo**

According to Caovilla et al.'s^19^ criteria.

Biological calibration of ocular movements

A biological calibrator (visual stimulator) was used for ocular movement calibration.

Investigation of spontaneous (NE) and semi-spontaneous (NSE) nystagmus

A light bar (visual stimulator) was used to investigate spontaneous nystagmus.

**Investigation of saccadic movements**

A light bar (visual stimulator) was used for this test.

**Investigation of pendular tracking**

A light bar (visual stimulator) was used for this test.

**Investigation of optokinetic nystagmus**

A light bar (visual stimulator) was used for this test.

**Investigation of per-rotatory nystagmus**

A YOSHI pendular rotating chair was used for this test.

**Investigation of post-caloric nystagmus**

An air otocalorimeter was used to test post-caloric nystagmus.

**Assessment parameters**

According to Caovilla et al.'s^19^ guidelines, for evaluating parameters of interest in vestibular function.

**Analysis criteria**:

The interpretation of vestibular testing was done according to Ganança et al.'s parameters.^20^

**Statistical method**:

The analysis of vestibular test results was based on the following tests, given the nature of the variables:

• parametric: Student's t test, controlled by Levene's test21 to compare the means of two variables;
• non-parametric: The Mann-Whitney test^22^ to check associations between variables.

The confidence limit was 95%, based on the mean and standard deviation values of variables.

The level for rejecting the null hypothesis was 0.05 or 5% (a≤0.05); we highlighted the significant values.

The SPSS (Statistical Package for Social Sciences) software, version 13.0, was used for obtaining the results.

## RESULTS

Table 1 shows the sample distribution relating school performance and sex.

**Table 1:** Sample distribution according to sex and school performance (n=50).

Learning disability	Sex		Total
	Female	Male	
	15	16	31
No	48.4%	51.6%	100.0%
	8	11	19
	42.1%	57.9%	100.0%
	23	27	50
Total	46.0%	54.0%	100.0%

Table 2 shows the comparative analysis of the sample as percentages of the most frequent complaints related to school performance. The Mann-Whitney test applied to these differences revealed that only the complaint “dazedness” was statistically significant (p= 0.043).

**Table 2:** Comparative analysis of the sample as percentages of the most frequent complaints related to school performance (n= 50).

Learning disability	Most frequent complaints												Total, by complaint
	dizziness		dazedness		oscillation		vertigo		fluctuation		instability		
	with	without	with	without	with	without	with	without	with	without	with	without	
No	10	21	1	30	0	31	5	26	1	30	4	27	31
	32,3%	67,7%	3,2%	96,8%	0,0%	100,0%	16,1%	83,9%	3,2%	96,8%	12,9%	87,1%	100,0%
Yes	8	11	4	15	1	18	2	17	1	18	2	17	19
	42,1%	57,9%	21,1%	78,9%	5,3%	94,7%	10,5%	89,5%	5,3%	94,7%	10,5%	89,5%	100,0%
Total	18	32	5	45	1	49	7	43	2	48	6	44	50
	36,0%	64,0%	10,0%	90,0%	2,0%	98,0%	14,0%	96,0%	4,0%	96,0%	12,0%	88,0%	100,00
significance	0,077		0,043 *		0,201		0,583		0,724		0,804		

(The Mann-Whitney test for the difference between variables).

Table 3 presents a comparative analysis of the sample as percentages of the most frequent symptoms in school related to school performance. The Mann-Whitney test for the differences revealed that only the symptom “nausea” had a statistically significant relation (p= 0.007).

**Table 3:** Comparative analysis of the sample as percentages of the most frequent symptoms in school related to school performance (n= 50).

Learning disability	Most frequent symptoms in school												Total by sympto
	headache		anxiety		otalgia		vomiting		nausea		dizziness		
	with	without	with	without	with	without	with	without	with	without	with	without	
No	14	17	3	28	3	28	1	30	2	29	4	27	31
	45,2%	54,8%	9,7%	90,3%	9,7%	90,3%	3,2%	96,8%	6,5%	93,5%	12,9%	87,1%	100,0%
Yes	11	8	0	19	3	16	2	17	7	12	5	14	19
	57,9%	42,1%	0,0%	100%	15,8%	84,2%	10,5%	89,5%	36,8%	63,2%	26,3%	73,7%	100,0%
Total	25	25	3	47	6	44	3	47	9	41	9	41	50
	50,0%	50,0%	6,0%	94,0%	12,0%	88,0%	6,0%	94,0%	18,0%	82,0%	18,0%	82,0%	100,00
significance	0,387		0,166		0,523		0,296		0,007 *		0,236		

(The Mann-Whitney test for the difference between variables).

Table 4 presents a comparative analysis of the sample as percentages of abilities related to school performance. The Mann-Whitney test for the differences revealed that lacking the abilities of “skipping” (p=0.016) and “riding a bicycle” (p=0.001) were statistically significantly related to the study variables.

**Table 4:** Comparative analysis of the sample as percentages of abilities related to school performance (n= 50).

Learning disability	Abilities												Total by ability
	circle dance		skipping		ride a bicycle		ride a car		height		hopscotch		
	yes	no	yes	no	yes	no	yes	no	yes	no	yes	no	
	6	25	1	30	0	31	1	30	1	30	1	30	31
	19,4%	80,6%	3,2%	96,8%	0,0%	100,0%	3,2%	96,8%	3,2%	96,8%	3,2%	96,8%	100,0%
Yes	3	16	5	14	6	13	1	18	0	19	1	18	19
	15,8%	84,2%	26,3%	73,7%	12,0%	68,4%	5,3%	94,7%	0,0%	100,0%	5,3%	94,7%	100,0%
Total	9	41	6	44	6	44	2	48	1	49	2	48	50
	18,0%	82,0%	19,4%	88,0%	12,0%	88,0%	4,0%	96,0%	2,0%	98,0%	4,0%	96,0%	100,00
significance	0,753		0,016 *		0,001 *		0,724		0,434		0,724		

(The Mann-Whitney test for the difference between variables).

Table 5 presents a comparative analysis of the sample as percentages of learning disabilities related to school performance. The Mann-Whitney test for the differences showed that “reading” difficulties (p<0.001) and “copying” (p<0.001) had a statistically significant relation in the variables.

**Table 5:** Comparative analysis of the sample as percentages of learning disabilities related to school performance (n= 50).

Learning disability	Learning disabilities								Total by disability
	reading		copying		concentration		blurred vision		
	yes	no	yes	no	yes	no	yes	no	
No	10	21	4	27	6	25	0	31	31
	32,3%	67,7%	12,9%	87,1%	19,4%	80,6%	0,0%	100,0%	100,0%
Yes	18	1	14	5	1	18	1	18	19
	94,7%	5,3%	73,3%	26,3%	5,3%	94,7%	5,3%	94,7%	100,0%
Total	28	22	18	32	7	43	1	49	50
	56,0%	44,0%	36,0%	64,0%	14,0%	86,0%	2,0%	98,0%	100,00
significance	<0,001 *		<0,001 *		0,168		0,201		

(The Mann-Whitney test for the difference between variables).

Table 6 shows the result of Student's t test controlled by Levene's test for the Equality of Variances to investigate possible differences between parametric variable means for the oculomotor calibration parameters latency, velocity and accuracy.

**Table 6:** Distribution of the sample relating calibration oculomotor parameters and school performance (n=50).

Variable	difficulty	n	Mean	Standard deviation	Significance (p)
latency_D_1	No	31	143,94	84,99	0,360
	Yes	19	165,82	74,41	
velocity_D_1	No	31	145,85	66,97	0,694
	Yes	19	138,88	47,46	
accuracy_D_1	No	31	85,26	23,34	0,428
	Yes	19	79,47	27,29	
latency_E_1	No	31	156,39	79,21	0,213
	Yes	19	184,63	72,53	
velocity_E_1	No	31	150,58	65,17	0,580
	Yes	19	140,52	56,19	
	No	31	86,83	16,01	
	Yes	19	86,74	18,69	

**Figure 1:**
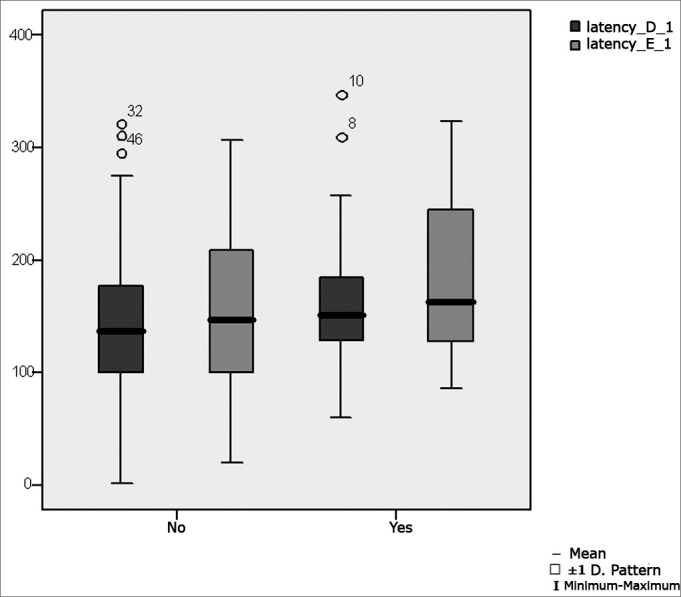
Boxplot for the right and left calibration oculomotor parameter (latency), per group. (Student's t test, controlled by Levene's test for equality of variances, for the means of parametric variables of interest).

Figure 1 presents the statistical investigation for the calibration oculomotor parameter latency, in boxplot format.

**Figure 2:**
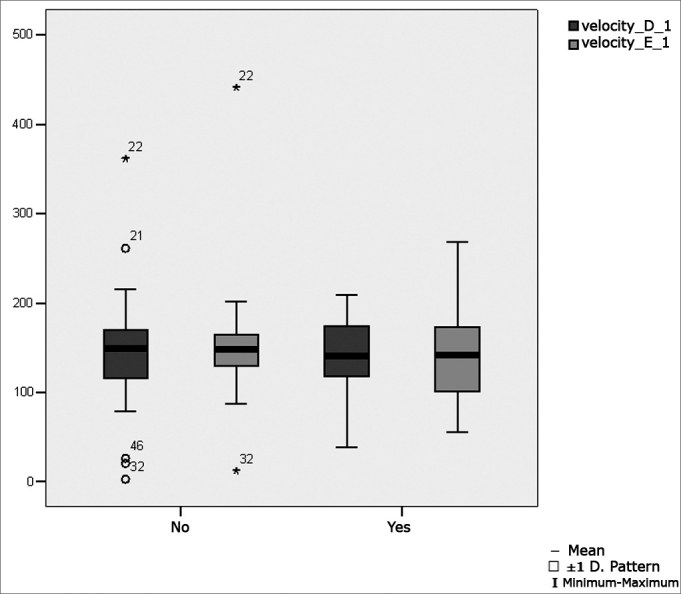
Boxplot for the right and left calibration oculomotor parameter (velocity), per group. (Student's t test, controlled by Levene's test for equality of variances, for the means of parametric variables of interest).

Figure 2 presents the statistical investigation for the calibration oculomotor parameter velocity, in boxplot format.

**Figure 3:**
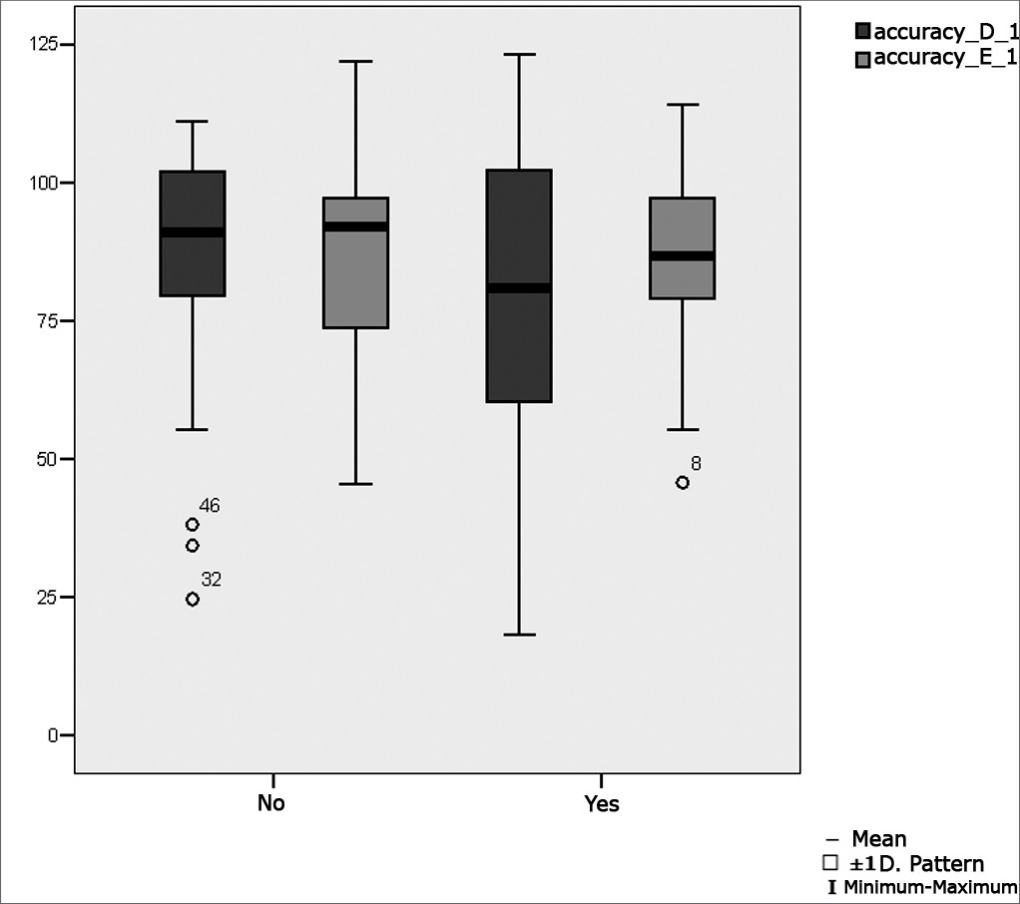
Boxplot for the right and left calibration oculomotor parameter (accuracy), per group. (Student's t test, controlled by Levene's test for equality of variances, for the means of parametric variables of interest).

Figure 3 presents the statistical investigation for the calibration oculomotor parameter accuracy, in boxplot format.

Table 7 shows the result of Student's t test controlled by Levene test for the Equality of Variances to investigate possible differences between parametric variable means for the oculomotor saccadic movement calibration parameters latency, velocity and accuracy.

**Table 7:** Distribution of the sample relating oculomotor saccadic movement calibration parameters and school performance (n=50).

Variable	difficulty	n	Mean	Standard deviation	Significance (p)
latency D 2	No	31	188,33	63,00	0,677
	Yes	19	196,39	71,01	
velocity D 2	No	31	93,91	32,73	0,144
	Yes	19	107,72	30,51	
accuracy D 2	No	31	92,09	26,32	0,369
	Yes	19	98,68	22,47	
latency E 2	No	31	187,31	55,10	0,544
	Yes	19	177,86	49,55	
velocity E 2	No	31	100,27	32,17	0,458
	Yes	19	107,25	31,78	
accuracy E 2	No	31	98,28	28,37	0,053
	Yes	19	118,57	37,98	

**Figure 4:**
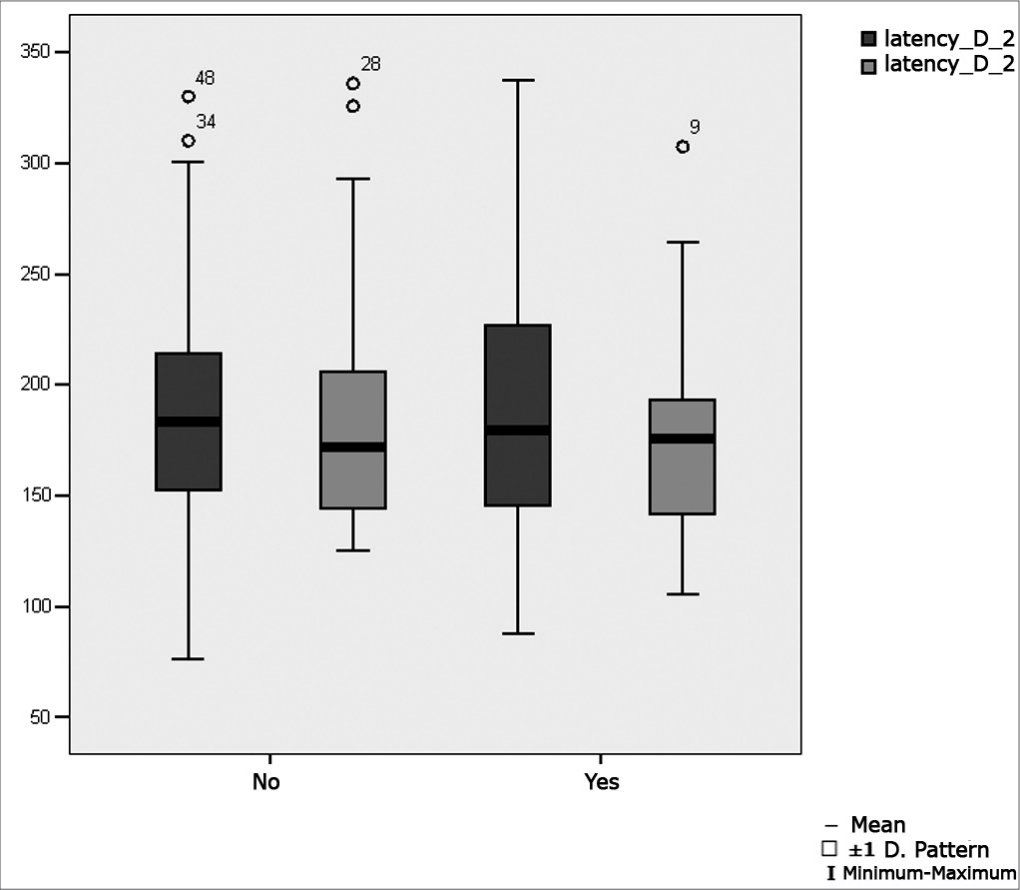
Boxplot for the right and left saccadic movement oculomotor parameter (latency), per group. (Student's t test, controlled by Levene's test for equality of variances, for the means of parametric variables of interest).

Figure 4 shows the statistical investigation for the oculomotor saccadic movement parameter latency in boxplot format.

**Figure 5:**
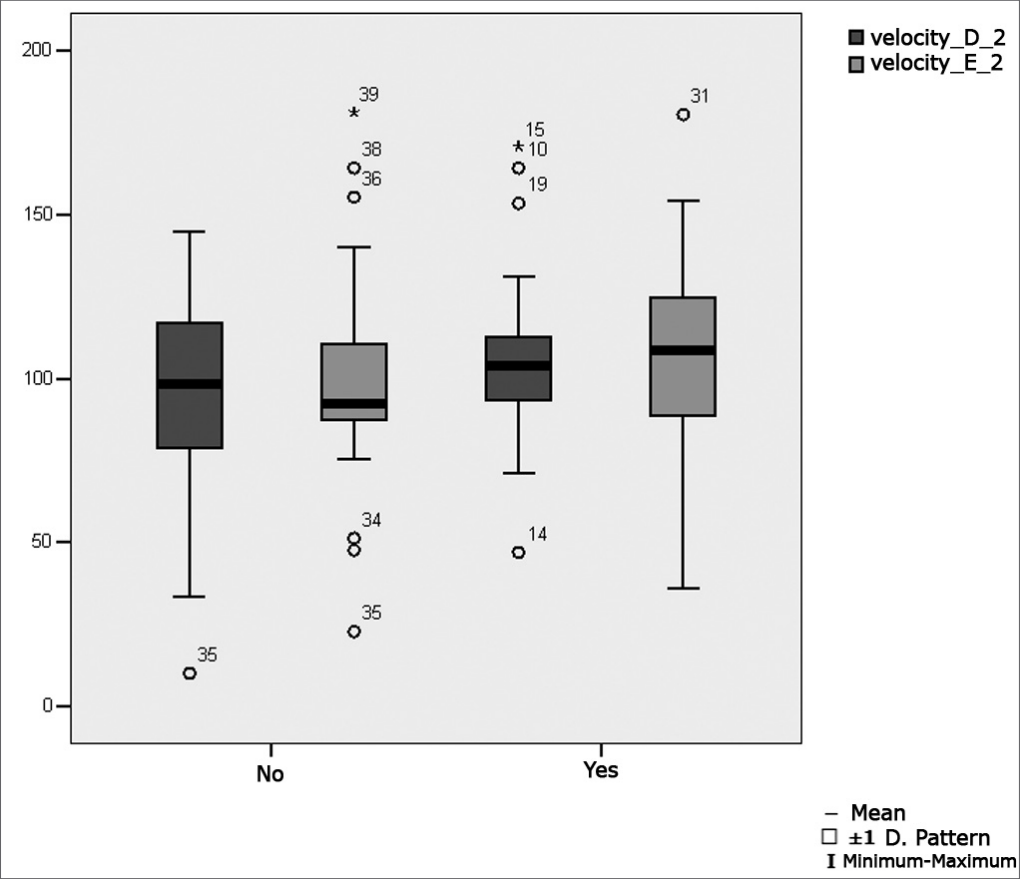
Boxplot for the right and left saccadic movement oculomotor parameter (velocity), per group. (Student's t test, controlled by Levene's test for equality of variances, for the means of parametric variables of interest).

Figure 5 shows the statistical investigation for the oculomotor saccadic movement parameter velocity in boxplot format.

**Figure 6:**
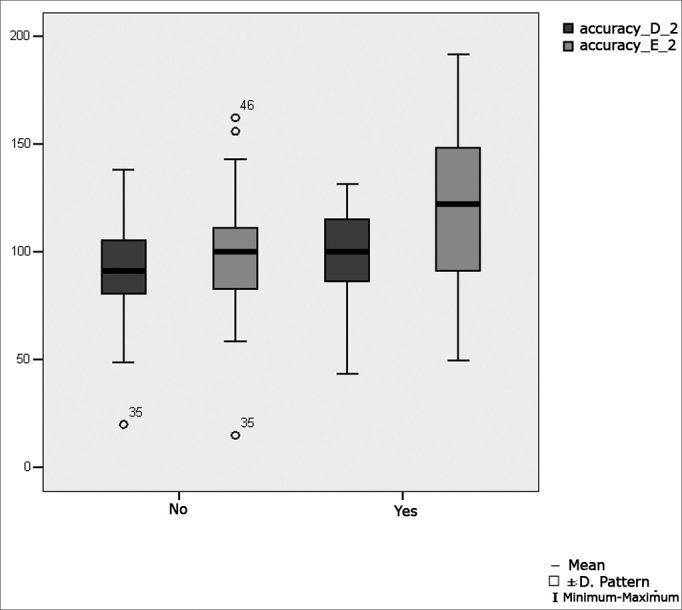
Boxplot for the right and left saccadic movement oculomotor parameter (accuracy), per group. (Student's t test, controlled by Levene's test for equality of variances, for the means of parametric variables of interest).

Figure 6 shows the statistical investigation for the oculomotor saccadic movement parameter accuracy in boxplot format.

Table 8 presents the results of Student's t test controlled by Levene test for the Equality of Variances to investigate possible differences between parametric variable means for pendular tracking gains at 20Hz, 40Hz and 80Hz.

**Table 8:** Distribution of the sample relating pendular tracking gains and school performance (n=50).

Variable	difficulty	n	Mean	Standard deviation	Significance (p)
gain_20Hz	No	31	0,86	0,26	0,408
	Yes	19	0,79	0,25	
gain_40Hz	No	31	1,00	0,22	0,722
	Yes	19	0,97	0,21	
gain_80Hz	No	31	0,84	0,19	0,571
	Yes	19	0,88	0,21	

**Figure 7:**
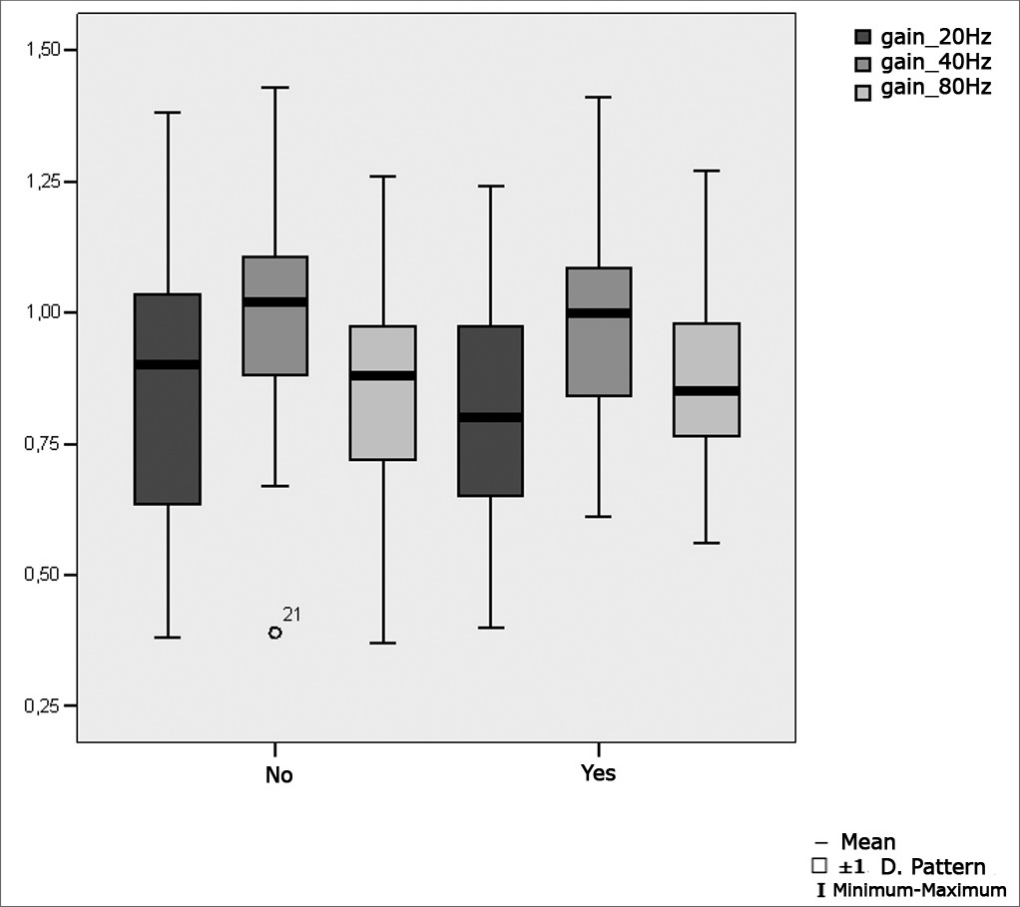
Boxplot for the parameter gain and pendular tracking at 20Hz, 40Hz and 80Hz, por group. (Student's t test, controlled by Levene's test for equality of variances, for the means of parametric variables of interest).

Figure 7 shows the statistical investigation for pendular tracking gains at 20Hz, 40Hz and 80Hz in boxplot format.

Table 9 presents the results of Student's t test controlled by Levene test for the Equality of Variances to investigate possible differences between parametric variable means for nystagmus directional preponderance in the optokinetic test.

**Table 9:** Distribution of the sample relating nystagmus directional preponderance in the optokinetic test and school performance (n=50).

Variable	difficulty	n	Mean	Standard deviation	Significance (p)
OPTO_PDN	No	31	6,21	5,44	0,840
	Yes	19	5,91	4,38	
VACLD	No	31	10,99	2,57	0,676
	Yes	19	11,29	2,28	
VACLE	No	31	10,91	2,40	0,877
	Yes	19	10,81	2,12	

**Figure 8:**
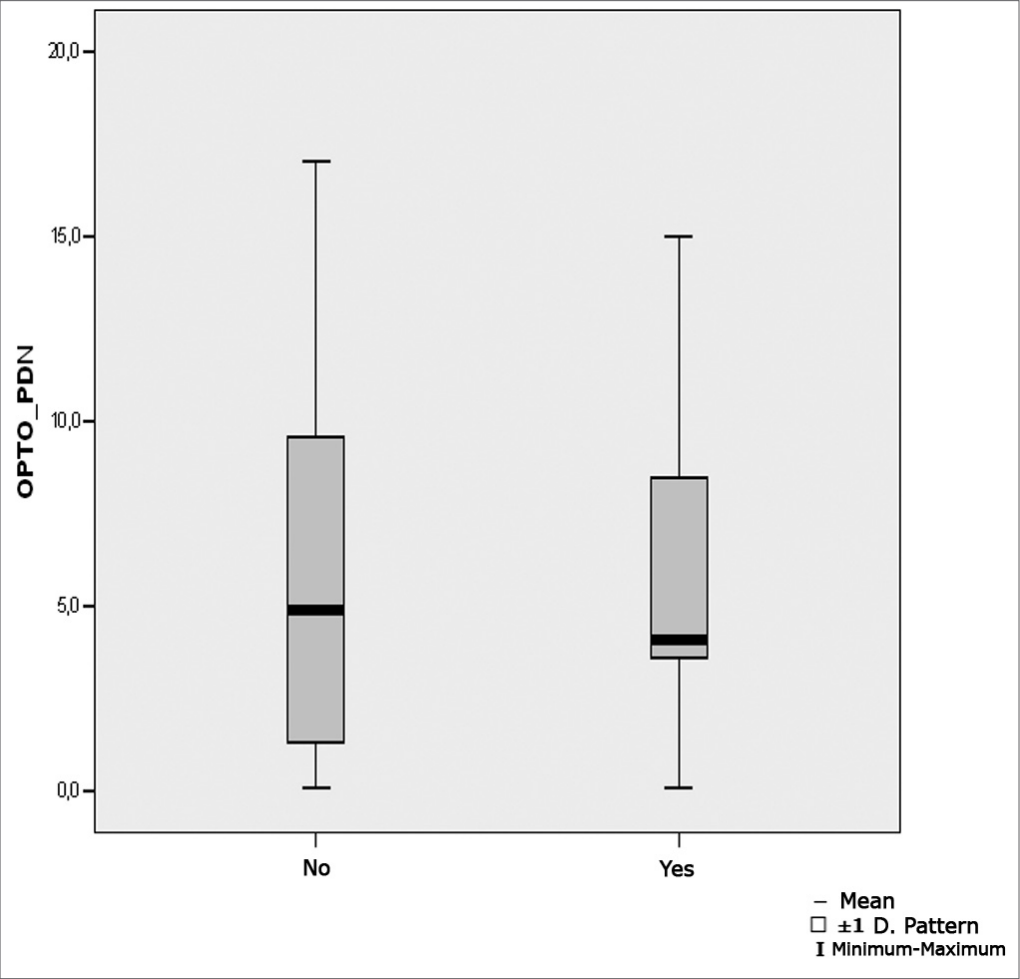
Boxplot for the directional preponderance of nystagmus in the optokinetic test, per group. (Student's t test, controlled by Levene's test for equality of variances, for the means of parametric variables of interest).

Figure 8 show the statistical investigation for nystagmus directional preponderance in the optokinetic test in boxplot format.

Table 10 presents the results of Student's t test controlled by Levene test for the Equality of Variances to investigate possible differences between parametric variable means for nystagmus directional preponderance in the decreasing pendular rotating test for the lateral semicircular canals (PDN L), posterior semicircular canals (PDN P) and superior semicircular canals (PDN S).

**Table 10:** Distribution of the sample relating nystagmus directional preponderance in the decreasing pendular rotating test and school performance (n=50).

Variable	difficulty	n	Mean	Standard deviation	Significance (p)
PDN_L	No	31	13,83	7,58	0,034 *
	Yes	19	9,12	7,16	
PDN_P	No	31	14,22	7,72	0,672
	Yes	19	13,32	6,54	
PDN_S	No	31	12,06	7,57	0,372
	Yes	19	14,22	9,16	

**Figure 9:**
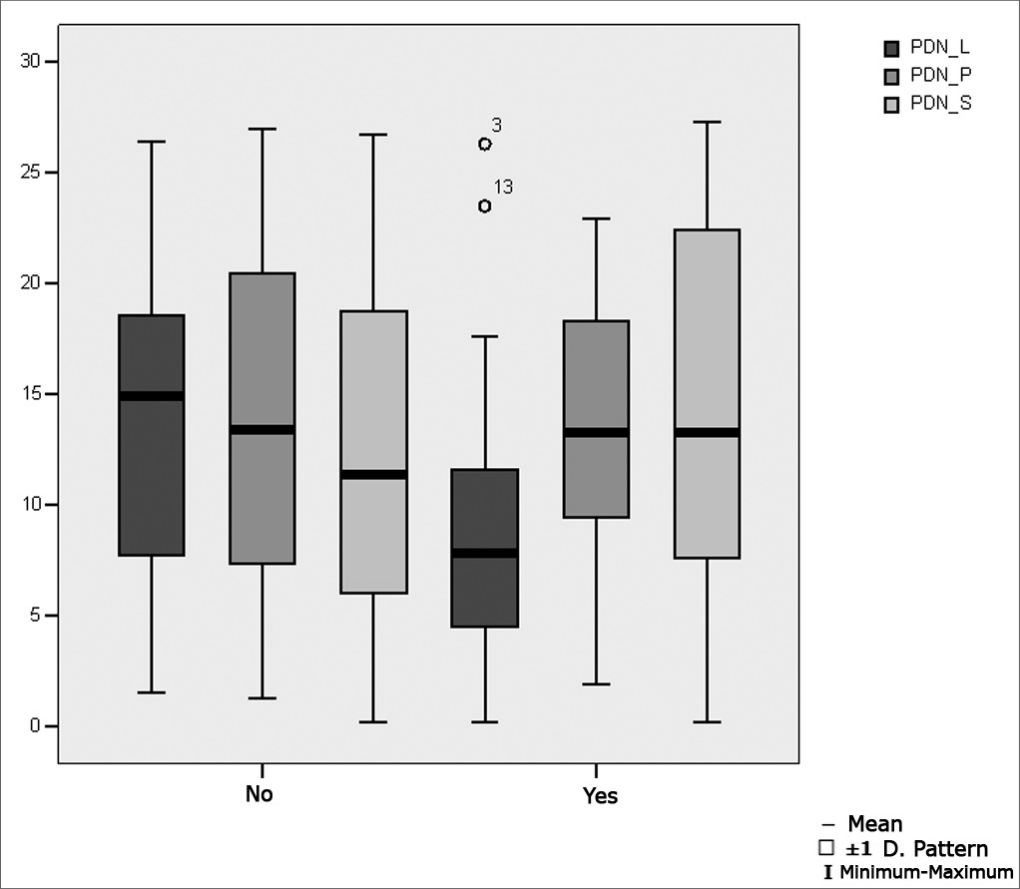
Boxplot for the directional preponderance of nystagmus in the decreasing pendular rotatory test, per group. (Student's t test, controlled by Levene's test for equality of variances, for the means of parametric variables of interest).

Figure 9 shows the statistical investigation for nystagmus directional preponderance in the decreasing pendular rotating test for the lateral semicircular canals (PDN L), posterior semicircular canals (PDN P) and superior semicircular canals (PDN S), in boxplot format.

Table 11 presents the results of Student's t test controlled by Levene test for the Equality of Variances to investigate possible differences between parametric variable means for the nystagmus slow component angular velocity in the caloric test at 42°C and at 18°C in both ears.

**Table 11:** Distribution of the sample relating the nystagmus slow component angular velocity in the caloric test at 42°C and at 18°C in both ears and school performance (n=50).

Variable	difficulty	n	Mean	Standard deviation	Significance (p)
PC	No	31	16,15	7,55	0,053
	Yes	19	22,55	12,42	
D 42°.C	No	31	9,17	3,12	0,477
	Yes	19	10,20	5,69	
E 42°.C	No	31	10,56	3,58	0,760
	Yes	19	11,02	6,81	
D 18°.C	No	31	15,59	7,44	0,041 *
	Yes	19	20,77	9,93	
E 18°.C	No	31	13,65	7,06	0,189
	Yes	19	16,88	10,05	

**Figure 10:**
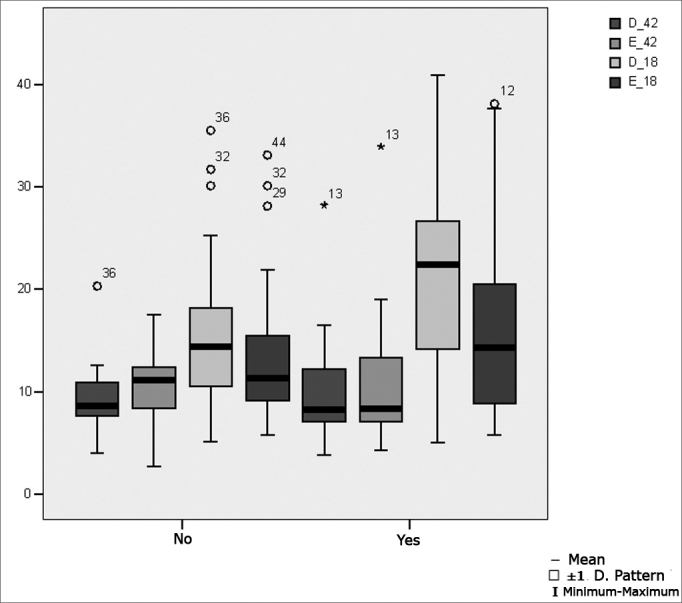
Boxplot for the right and left caloric test at 18°C and 42°C, per group. (Student's t test, controlled by Levene's test for equality of variances, for the means of parametric variables of interest).

Figure 10 shows the statistical investigation for the nystagmus slow component angular velocity in the caloric test at 42°C and at 18°C in both ears, in boxplot format.

Table 12 presents the sample distribution relating final diagnosis percentages to school performance. The Mann-Whitney test for the differences revealed that there is a statistically significant relation between the variables that were investigated (p= 0.007).

**Table 12:** Distribution of final diagnosis percentages related to school performance (n=50).

Final diagnosis						
Learning disability	EVN	SVPI	SVPIB	SVPID	SVPIE	Total
No	23	0	5	2	1	31
	74,2%	0,0%	16,1%	6,5%	3,2%	100,0%
Yes	6	2	6	4	1	19
	31,6%	10,5%	31,6%	21,1%	5,3%	100,0%
Total	29	2	11	6	2	50
	58,0%	4,0%	22,0%	12,0%	4,0%	100,00

Key:

EVN = Normal vestibular examination;

SVPI = Irritative peripheral vestibular syndrome;

SVPIB = Bilateral irritative peripheral vestibular syndrome;

SVPID = Right irritative peripheral vestibular syndrome;

SVPIE = Left irritative peripheral vestibular syndrome.

## DISCUSSION

Our results show the sample distribution related to school performance percentages and gender. We found similarity (p=0.668) between the variables we investigated, with no significant gender differences.

We found that 31 of 50 children (62.0%) did not report leaning disabilities, and that 19 (38.0%) reported learning disabilities.

Our data were similar to those of Farias et al.5 in their assessment of 103 children with and with not learning disabilities, of which 60 children (58.2%) presented no learning disabilities and 43 children (41.7%) presented learning disabilities.

Polity's,^1^ Undheim's,^2^ and Mathes and Denton's^3^ papers should be taken into account; they state that the etiology of learning disabilities is varied, and may involve organic factors. According to these authors, in most cases there is an interrelationship between intellectual/cognitive, emotional and organic factors, including insufficient or inappropriate education.

Schirmer et al.^4^ discussed the existence of neurological factors in language and learning disabilities, and underlined the importance of neurobiological developments on language and learning processes.

We present the percentages for common general complaints in our sample; we noted that the complaint “dazedness” was statistically significant (p=0.043) in separating children with and without learning disabilities.

Our data confirm Ganança and Caovilla's^7^ position that the vestibular proprioceptive system controls body position, eye movement and spatial perception, all of which these authors consider essential for language acquisition and learning. These findings are also similar to those of Campos et al.;^6^ these authors concluded that the vestibular system has a significant influence on child development, as this system - together with the central nervous system - controls body position, eye movement and spatial perception.

We describe for our sample the percentages of the most common symptoms in school. There was a statistically significant (p=0.007) relation between the symptom “nausea” and children with learning disabilities.

Based on this analysis we found that 25 (50.0%) of 50 children reported the symptom headache. This symptom was frequent, although there was no statistically significant relation between this and other variables.

These findings suggest, as Campos et al.^6^ have described, that there may be involvement of the vestibular system evidenced by nausea, headache and ill-defined malaise.

Lavinsky et al.^9^ described criteria for identifying labyrinthic dysfunction in children, including: nausea, unexplained vomiting, oral and written language acquisition disabilities and a history of migraine.

Caovilla et al.8 suggest vestibular testing in every child with a strong clinical suspicion of vestibular dysfunction, even though an accurate description of symptoms from children or their parents may not be forthcoming.

Data comparing the abilities of children with and with no learning disabilities revealed statistically significant relations between the variables aptitude with the games skipping (p=0.016) and bicycle riding (p=0.001).

Of the 50-children sample 41 children (82.0%) did not like to play circle dancing; 16 of them (39.0%) presented learning disabilities. Of the 50-children sample 44 children (88.0%) did not like skipping; 14 of them (31.8%) presented learning disabilities. Of the 50-children sample 44 children (88.0%) did not like bicycle riding; 13 of them (29.5%) presented learning disabilities.

These findings are similar to Caovilla et al.'s^8^ results; this author concluded that difficulties in carrying out coordinated movement and imprecise spatial positioning of oneself and of objects affect learning. Lack of aptitude for certain physical movements and distorted perception of spatial relations impairs contact between children and the environment, which affects negatively their physical and psychical development.

According to Formigoni,^10^ these children are frequently agitated, seeking bodily positions that offer comfort and safety; this leads to lack of concentration and dispersion. These children may not like to play, to ride bicycles, to walk on top of walls or curbs, to skip or play hopscotch.

A comparative percentage analysis of learning disabilities related to school performance showed that “reading” (p<0.001) and “copying” (p<0.001) difficulties had a statistically significant relation.

Of the 50-children sample, 28 children (56.0%) reported reading difficulties; 18 of them (64.2%) presented learning disabilities. Of the 50-children sample, 18 (36.0%) reported copying difficulties; 14 of them (77.7%) presented learning disabilities.

These data are similar to Hoyt's^14^ findings; this author states in his paper that ocular movements needed for reading require alternating saccadic movements and fixation periods. Full integration between the vestibular apparatus and saccadic movements are essential.

We found that ocular movement calibration means were within normal limits in the digital vectonystagmographic assessment, according to Ganança et al.'s^20^ reference values for the parameters latency and velocity, in children with and with no learning disabilities. We found values below normal limits (Ganança et al.20), however, for the parameter accuracy (79.4%) in children with learning disabilities.

These data corroborate Frank and Levinson's^11^ hypothesis that vestibular dysfunction could interfere on sequential ocular fixation, which is necessary for reading.

Our findings are also similar to those in other studies, such as those by Horak et al.^13^ on vestibular function in children with and with no reading and writing learning disabilities; this author found altered vestibular reflexes in 20% of children with learning disabilities. According to Hoyt,14 ocular movements needed for reading require alternating saccadic movements and fixation periods. Full integration between the vestibular apparatus and saccadic movements are essential.

Activities that require healthy oculomotor function and vestibular integration include following the teacher within one's visual field in the classroom, making copies, transcribing lessons written on a blackboard, reading books, writing and concentrating.

We found that caloric test means were within normal limits in the digital vectonystagmographic assessment, according to Ganança et al.'s^20^ reference values for warm thermal stimulation (42°C), in children with and with no learning disabilities. We found values above normal limits (Ganança et al.^20^), however, for cold thermal stimulation (18°C) in children with learning disabilities, with a statistically significant relation between variables (p= 0.041).

Our data revealed vestibular dysfunction related to labyrinthic excitation, which led to vestibular hyperactivity.

According to Campos et al.,^6^ child vestibular dysfunction may considerably affect communication abilities, the psychological state and school performance; it should be borne in mind that poor school performance may be a valuable indicator of labyrinth disease.

The final diagnosis in our school performance sample revealed a high rate of normal vestibular examinations (74.2%) in children with no learning disabilities; a low rate of normal examinations was found in children with learning disabilities (31.6%). All of the vestibular alterations were of a bilateral or unilateral irritative peripheral origin, totaling 68.4% in children with learning disabilities and 25.8% for children with no learning disabilities. Our data revealed a statistically significant relation between the variables (p= 0.007).

Our results are similar to those of Ganança.^15^ This author assessed 64 children with language disabilities and no complaints of dizziness, and found a high incidence of peripheral topodiagnostic vestibular alterations.

Quirós^12^ found that vestibular system alterations in children could lead to speech disorders, and underlined the need for carefully evaluating school-age children for early intervention programs, if needed.

Deep knowledge about school performance and it alterations add value to research in speech therapy, and clarify cloudy aspects that hinder appropriate interventions.

An accurate correlation between learning disabilities and the vestibular system is important, and raises the need for further research to confirm our data and to clarify doubts for which there are currently no answers.

## CONCLUSION

We concluded that a complaint of dazedness and the symptom nausea had a statistically significant relation with reading and copying disabilities. Our data show a statistically significant relation between vestibular findings and learning disabilities. All of the vestibular findings were of peripheral irritative origin.

## References

[1] Polity E (2003). Dificuldade de Ensinagem: Que história é essa?. Fonoaudiologia Atual..

[2] Undheim AM (2003). Dyslexia and psychosocial factors.. A follow-up study of young Norwegian adults with a history of dyslexia in childhood. Nord J Psychiatry..

[3] Mathes PG, Denton CA (2002). The prevention and identification of reading disability.. Semin Pediatr Neurol.

[4] Schirmer CR, Fontoura DR, Nunes ML (2004). Distúrbios da aquisição da linguagem e da aprendizagem.. J Pediatr.

[5] Farias LS, Toniolo IF, Coser PL (2004). P300: avaliação eletrofisiológica da audição em crianças sem e com repetência escolar.. Rev Bras Otorrinolaringol.

[6] Campos MI, Ganança FF, Caovilla HH, Ganança MM (1996). Prevalência de sinais de disfunção vestibular em crianças com vertigem e/ou outros tipos de tontura.. RBM-ORL.

[7] Ganança MM, Caovilla HH, N Caldas, S Caldas, T Sih (1999). Otologia e audiologia em pediatria..

[8] Caovilla HH, Ganança MM, Munhoz MSL, Silva MLG, Ganança FF, Frazza MM, MLG Silva, MSL Munhoz, MM Ganança, HH Caovolla (2000). Quadros clínicos otoneurológicos mais comuns..

[9] Lavinsky L, Abelin CA, D'Avila C, Lavinsky M, N Caldas, S Caldas, T Sih (1999). Otologia e audiologia em pediatria..

[10] Formigoni LG, MM Ganança (1998). Vertigem tem cura?.

[11] Frank J, Levinson H (1973). Dysmetric dyslexia and dyspraxia.. J Am Acad Child Psychiatry.

[12] Quirós de JB (1976). Diagnosis of vestibular disorders in learning disabled.. J Learn Desabil.

[13] Horak FG, Shumway-Cook A, Crowe TK, Black FO (1988). Vestibular function and motor proficiency of children with impaired hearing, or with learning disability and motor impairment.. Dev Med Child Neurol.

[14] Hoyt CS (1999). Visual training and reading.. Am Orthopt J.

[15] Ganança MM (1989). Da vestibulometria em crianças com distúrbio de linguagem. [Tese de Doutorado].

[16] Glorig A, Davis H (1961). Age, noise and hearing loss.. Ann Otol (St. Louis).

[17] Mangabeira Albernaz P, Mangabeira Albernaz PL, Mangabeira Albernaz LG, Mangabeira Albernaz Filho P (1981). Otorrinolaringologia prática..

[18] Mangabeira Albernaz PL, Ganança MM, Caovilla HH, Ito YI, Novo NF, Juliano I (1986). Aspectos Clínicos e Terapêuticos das Vertigens.. Acta WHO.

[19] Caovilla HH, Ganança MM, Munhoz MSL, Silva MLG (1999). Equilibriometria Clínica..

[20] Ganança CF, Souza JAC, Segatin LA, Caovilla HH, Ganança MM (2000). Limites de normalidade dos parâmetros de avaliação a vectonistagmografia digital neurograff.. Acta AWHO.

[22] Callegari-Jacques SM (2003). Bioestatística: princípios e aplicações..

